# Effect of adding preoperative serratus anterior plane block to intercostal nerve block with liposomal bupivacaine on postoperative pain in patients undergoing video-assisted thoracoscopic lung resection: study protocol for a prospective randomized controlled trial

**DOI:** 10.3389/fmed.2026.1948798

**Published:** 2026-10-13

**Authors:** Huiqiu Liang, Qin Cui, Qun Wang, Hongzhang Ding, Yu Zhou, Zhikai Ling, Maolin Ran, Lijun Tang

**Affiliations:** 1 Shanghai Ninth People’s Hospital, Shanghai Jiao Tong University School of Medicine, Shanghai, China; 2 Shanghai General Hospital, Shanghai Jiao Tong University School of Medicine, Shanghai, China

**Keywords:** intercostal nerve block, liposomal bupivacaine, postoperative pain, serratus anterior plane block, study protocol, video-assisted thoracoscopic surgery

## Abstract

**Background:**

Effective postoperative pain management is fundamental to enhanced recovery after video-assisted thoracoscopic surgery (VATS). Previous studies have demonstrated that both serratus anterior plane block (SAPB) and intercostal nerve block (ICNB) effectively alleviate postoperative pain. However, data on the combination of these two techniques using liposomal bupivacaine, a sustained-release local anesthetic formulation, remain limited. Accordingly, this randomized controlled trial aims to compare the postoperative analgesic efficacy of combined preoperative SAPB and intraoperative ICNB vs. sham SAPB plus active ICNB in patients undergoing VATS lung resection, with liposomal bupivacaine administered for both blocks.

**Methods:**

This single-center randomized controlled trial will enroll 98 patients scheduled for video-assisted thoracoscopic lung resection to compare the postoperative analgesic efficacy of combined preoperative SAPB and intraoperative ICNB vs. sham SAPB plus intraoperative ICNB, with liposomal bupivacaine administered for both nerve blocks. The primary outcome is the VAS (0–10) pain score with movement at 24 h postoperatively; secondary outcomes include cumulative sufentanil consumption within 48 h and the number of patient-controlled intravenous analgesia (PCIA) demands.

**Discussion:**

This trial will provide the first head-to-head evidence comparing the analgesic efficacy of combined SAPB and ICNB vs. sham SAPB plus ICNB using liposomal bupivacaine in VATS lung resection. If the combination strategy demonstrates superior analgesic efficacy, it may establish a more effective multimodal analgesic regimen for postoperative pain management in thoracic surgery. These findings are expected to inform clinical practice and guide the development of future perioperative analgesic protocols.

**Clinical Trial Registration:** https://www.chictr.org.cn/showproj.html?proj=295245, identifier ChiCTR2500112224.

## 1 Introduction

Thoracic surgery is currently performed predominantly via minimally invasive video-assisted thoracoscopic approaches. Despite reduced surgical trauma, patients commonly experience moderate to severe postoperative pain, rendering optimal analgesia essential (1, 2). Postoperative pain is an acute, time-limited phenomenon that typically emerges immediately following surgical intervention and generally resolves within 3–7 days. It is particularly prevalent after thoracic procedures. Inadequate early pain control may predispose certain patients to chronic postsurgical pain (CPSP) (3). Consequently, effective multimodal analgesia not only alleviates patient discomfort but also mitigates complications such as atelectasis and postoperative pulmonary infection, accelerates functional recovery, reduces hospital length of stay, enhances patient satisfaction, and yields broader societal benefits (4, 5). Postoperative analgesia following thoracic surgery typically employs a multimodal strategy encompassing preoperative regional nerve blockade, opioids, intravenous patient-controlled analgesia, and oral agents (6, 7). Although thoracic epidural and paravertebral blockade provide effective analgesia, epidural use has declined with the widespread adoption of video-assisted thoracoscopic surgery, reflecting concern regarding technical complexity and the risk of catheter-related infection (8–11). Emerging evidence suggests that recently developed fascial plane blocks may provide an effective alternative; however, an optimal analgesic regimen for thoracic surgery remains to be established.

The serratus anterior plane block (SAPB), first described by Blanco in 2013, is a well-established regional anesthesia technique that provides analgesia to the anterolateral chest wall by blocking the lateral cutaneous branches of the intercostal nerves, as well as the long thoracic and thoracodorsal nerves. Its technical simplicity, favorable safety profile, and compatibility with real-time ultrasound guidance have contributed to its increasing popularity in thoracic surgery and as an adjunctive analgesic modality following breast surgery (12–15). The intercostal nerve block (ICNB), typically performed by the surgeon under direct thoracoscopic visualization before wound closure, offers an alternative approach that effectively reduces incisional pain when used in conjunction with general anesthesia (16–18). Current evidence indicates that a single-injection ropivacaine nerve block provides analgesia for a limited duration, typically reported as approximately 4–6 h, although the actual duration varies depending on block technique, dose, concentration, and volume. In contrast, acute postoperative pain after thoracic surgery often persists for 2–3 days, highlighting a substantial gap between the duration of available analgesia and clinical needs. Consequently, prolonging postoperative analgesia is essential for effective pain management. Liposomal bupivacaine, a sustained-release local anesthetic formulation, provides analgesia for up to 72 h. Previous clinical studies have confirmed its efficacy and safety in improving postoperative pain control in obstetric, gynecologic, and abdominal surgical settings (19–22).

Recent evidence suggests that a single injection of liposomal bupivacaine for SAPB or ICNB alone may provide sufficient analgesia, with mean VAS scores below 2 lasting up to 72 h after VATS (23). However, these findings derive predominantly from single-arm or retrospective studies with inherent selection bias. To date, no head-to-head randomized controlled trial has directly compared combined SAPB plus ICNB vs. sham SAPB plus ICNB using liposomal bupivacaine in this patient population. The incremental analgesic benefit of adding preoperative SAPB to ICNB therefore remains unclear. If the combined strategy fails to demonstrate superiority over ICNB alone, this would support limiting the procedure to a single block, thereby reducing procedural time, puncture-related risks, and healthcare costs. Conversely, if superiority is established, it could define a more effective standard of care. Accordingly, this head-to-head comparison is clinically warranted.

However, current evidence regarding the combination of SAPB with ICNB remains limited (24). Available studies have not identified major safety signals with SAPB or ICNB individually; however, the safety of the combined SAPB plus ICNB approach has not been definitively established. Furthermore, as liposomal bupivacaine is a novel, long-acting local anesthetic formulation, this randomized controlled trial will compare the analgesic efficacy of liposomal bupivacaine SAPB plus ICNB vs. sham SAPB plus ICNB in patients undergoing video-assisted thoracoscopic lung resection. The overarching objective is to establish an optimal multimodal analgesic strategy for thoracic surgery.

## 2 Methods and analysis

### 2.1 Study design

This trial is a prospective, single-center, randomized clinical study designed in accordance with the SPIRIT reporting guidelines (25). Participants will be randomly assigned (1:1) to either the combined block group (Group C: SAPB plus ICNB) or the intercostal nerve block alone group (Group S: sham SAPB plus ICNB). This study will be conducted at Shanghai General Hospital, a tertiary grade-A hospital affiliated with Shanghai Jiao Tong University School of Medicine, Shanghai, China. The SPIRIT checklist of enrolment, interventions, and assessment is presented in Table 1, and the trial procedure is illustrated in Figure 1.

**Table 1:** Trial schedule.

Timepoint	Enrollment	Allocation	Post operation				
	−1 day	0 day	PACU	24h	48h	72h	Discharge
Enrollment							
Eligibility screen	×						
Informed consent	×						
Allocation		×					
Intervention							
Group C (SAPB + ICNB)		×					
Group S (sham SAPB + ICNB)		×					
Assessment							
VAS score (0–10)				×	×	×	
Opioid consumption		×	×	×	×		
PCA press counts				×	×		
PCA dosage (mL)				×	×		
Sufentanil dose		×	×	×	×		
Extubation time			×				
QoR-15						×	
Time to discharge							×
Complications			×	×	×	×	
Adverse events			×	×	×	×	

SAPB, serratus anterior plane block; ICNB, intercostal nerve block; VAS, visual analogue scale; PCA, patient-controlled analgesia; PACU, post-anesthesia care unit; QoR-15, quality of recovery-15.

**Figure 1:**
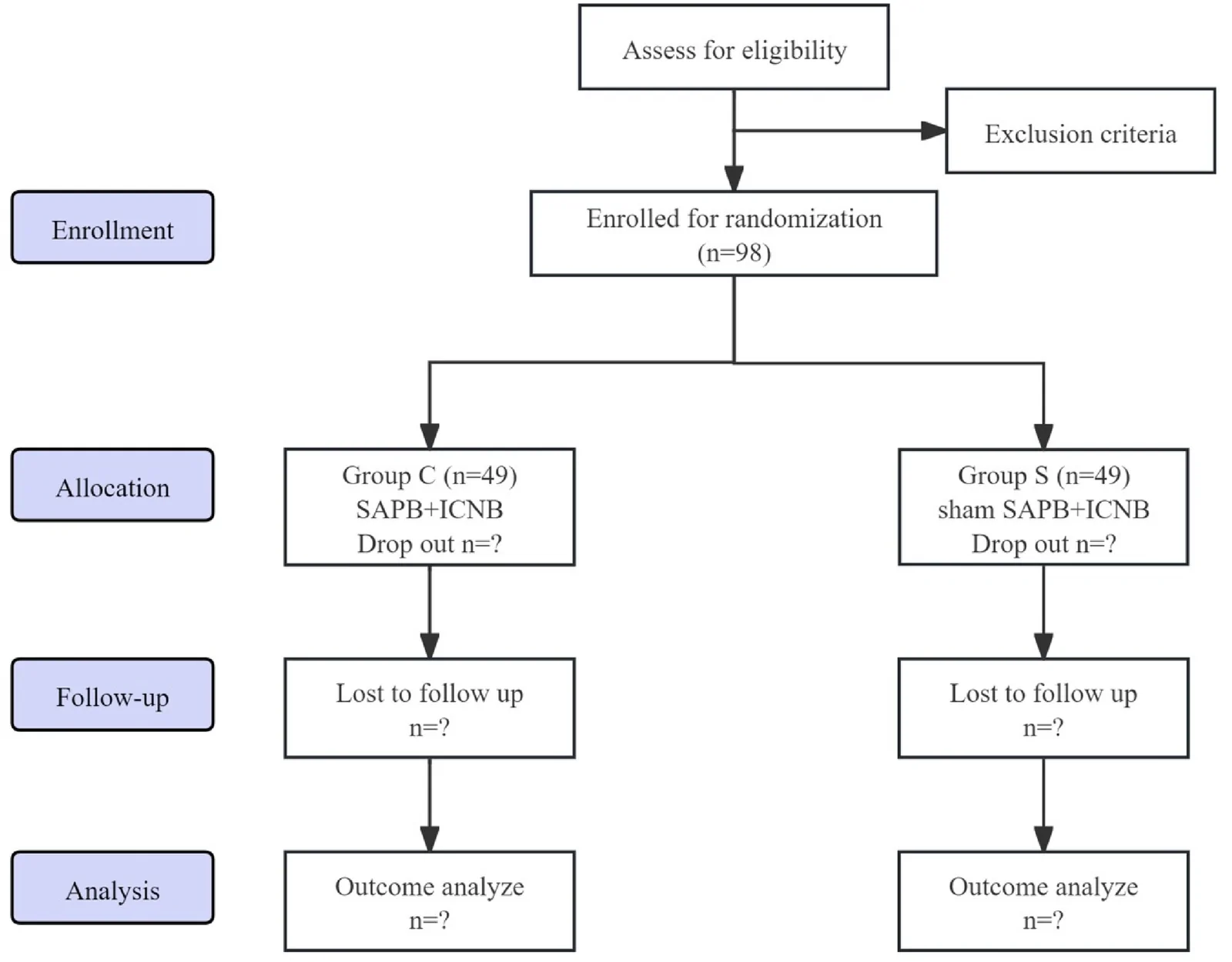
Flow chart of the trial. SAPB, serratus anterior plane block; ICNB, intercostal nerve block.

### 2.2 Objective

This study aims to compare the effect of SAPB plus ICNB vs. sham SAPB plus ICNB on postoperative pain in patients undergoing video-assisted thoracoscopic lung resection.

### 2.3 Recruitment and ethics

The study was approved by the Ethics Committee of Shanghai General Hospital and registered with the Chinese Clinical Trial Registry (ChiCTR2500112224; Principal investigator: Lijun Tang; registration date: November 11, 2025). The protocol was modified four times; the final version is V5.0/20260714. This trial does not involve biological sample collection. Patients scheduled for video-assisted thoracoscopic lung resection will be recruited and screened by the investigators one day prior to surgery. Those who meet the inclusion criteria will undergo informed consent procedures, during which they will be provided with detailed study information and asked to provide written informed consent.

### 2.4 Eligibility criteria

#### 2.4.1 Inclusion criteria

1. Age ≥18 years;
2. Scheduled for elective video-assisted thoracoscopic lung resection for the first time;
3. American Society of Anesthesiologists (ASA) physical status I–III;
4. Able to understand the study information and provide written informed consent.

#### 2.4.2 Exclusion criteria

1. Combination of severe cardiopulmonary, hepatic, or renal dysfunction;
2. Allergy to liposomal bupivacaine;
3. Preoperative coagulopathy or bleeding diathesis;
4. History of mental illness;
5. Inability to understand visual analogue scale (VAS).

#### 2.4.3 Withdrawal criteria

Only withdrawal of informed consent will result in exclusion from the study. Participants may withdraw consent at any time without affecting their clinical care.

### 2.5 Randomization and blinding

Participants will be randomized in a 1:1 ratio using a computer-generated random sequence with permuted block randomization (block size 8) to either the combined block group (Group C: SAPB + ICNB) or the intercostal nerve block alone group (Group S: sham SAPB + ICNB). The randomization list will be generated by a statistician not involved in the trial. Allocation will be concealed in sequentially numbered, opaque, sealed envelopes and opened in numerical order by an independent custodian who will have no other study role other than the secure storage of the envelopes and unblinding.

Blinding will be maintained for participants, investigators, and outcome assessors. Group C will receive an ultrasound-guided SAPB with liposomal bupivacaine prior to induction of anesthesia; Group S will receive an equal volume of 0.9% normal saline at the same site. Liposomal bupivacaine has a gradual onset of action. Immediately after the block procedure, patients are transferred to the operating room for induction of general anesthesia, before the onset of any perceptible sensory changes. A single anesthesiologist, not involved in subsequent data collection or analysis, will perform all SAPB/sham SAPB in the induction room. Participants are not informed of their group assignment before the procedure, and they are explicitly instructed not to discuss any sensations with the postoperative assessors.

Intraoperative anesthesiologists, surgeons, and postoperative assessors who are completely blinded to group allocation will remain blinded to group allocation throughout the study. All participants receive a standardized general anesthesia protocol (propofol/remifentanil) to minimize performance bias.

### 2.6 Intervention description

Group C will receive a preoperative ultrasound-guided deep SAPB with 10 mL of liposomal bupivacaine (Aihengping®; Jiangsu Hengrui Pharmaceuticals Co., Ltd., Lianyungang, China; 13.3 mg/mL, total dose 133 mg, undiluted). A high-frequency linear ultrasound transducer (6–13 MHz) will be placed in a sterile sheath and positioned at the mid-axillary line at the level of the fifth rib. The latissimus dorsi, serratus anterior, and teres major muscles will be identified, and Color Doppler will be used to locate the thoracodorsal artery as a landmark. A 22-gauge, 80-mm echogenic needle will be inserted using an in-plane approach from caudal to cranial direction. The target fascial plane is between the serratus anterior muscle and the rib (deep to the serratus anterior muscle), and correct injectate spread will be confirmed by real-time ultrasound visualization of fluid separating these two muscle layers. The 10-mL volume was selected based on previous anatomical and clinical studies demonstrating adequate spread within the deep serratus plane while minimizing excessive diffusion (26, 27). This will be followed by thoracoscopic ICNB performed by the surgeon under direct visualization prior to wound closure. The ICNB will be administered at three intercostal spaces adjacent to the surgical incision, specifically the space corresponding to the incision and one space immediately above and below. At each selected space, 3 mL of liposomal bupivacaine (13.3 mg/mL) will be injected, typically covering T3–T5 for most VATS procedures. The total planned dose of liposomal bupivacaine in Group C is 252.7 mg (SAPB 133 mg + ICNB 119.7 mg), which remains below the 266 mg maximum approved dose for local infiltration.

Group S will receive a sham SAPB with an equal volume (10 mL) of 0.9% normal saline before anesthesia induction, using identical ultrasound probe positioning, needle insertion approach, and injection technique as described for Group C. Real-time ultrasound will confirm injectate spread in the same fascial plane. This will be followed by ICNB with the same dose and technique as Group C.

In this study, liposomal bupivacaine will be used off-label for SAPB and ICNB. This off-label use is supported by published evidence on its safety in thoracic fascial plane blocks (23, 28) and pharmacokinetic data with doses up to 266 mg (29).

### 2.7 Anesthesia management

Both groups will follow an identical anesthetic protocol. Upon arrival in the operating room, standard monitoring will be initiated [electrocardiography, noninvasive blood pressure, and pulse oximetry (SpO_2_)]; a peripheral intravenous line will be placed; and a 20-gauge radial arterial catheter will be inserted under local anesthesia for continuous invasive blood pressure monitoring. Anesthesia will be induced with intravenous propofol 2 mg/kg, rocuronium 0.6 mg/kg, and sufentanil 0.25 μg/kg. Maintenance will be achieved with desflurane 4% to 6% or sevoflurane 1.5% to 2% in an oxygen/air mixture, titrated to maintain a bispectral index (BIS) of 50–60. A remifentanil infusion (0.1–0.3 *μ*g/kg/min) will be administered throughout surgery; systolic blood pressure and heart rate will be maintained within 25% of baseline using fluid boluses and vasoactive agents as needed.

Mechanical ventilation will be delivered in volume-controlled ventilation (VCV) mode. During two-lung ventilation, the settings will be: tidal volume 6–8 mL/kg of ideal body weight, respiratory rate 10–15 breaths/min, inspiratory-to-expiratory ratio 1:2, FiO₂ 0.6–1.0, and PEEP 5 cmH₂O. One-lung ventilation will be performed with a tidal volume of 4–6 mL/kg, with the respiratory rate adjusted to maintain end-tidal carbon dioxide (EtCO₂) between 35 and 45 mmHg and SpO_2_ ≥ 90%. If heart rate falls below 50 beats/min, atropine 0.5 mg will be administered intravenously. Episodes of systolic blood pressure below 75% of baseline will be treated with intravenous ephedrine 6 mg boluses. Thirty minutes before skin closure, sufentanil 10 μg will be given, followed by intravenous palonosetron 0.25 mg for prophylaxis of postoperative nausea and vomiting (PONV). Neuromuscular blockade will be reversed with sugammadex 200–400 mg based on train-of-four (TOF) monitoring. In this study, a multimodal analgesic regimen will be applied, comprising preoperative regional block for preemptive analgesia, intraoperative opioid administration, an additional nerve block before wound closure, and postoperative patient-controlled intravenous analgesia (PCIA) with sufentanil and antiemetics.

### 2.8 Follow-up

Postoperative follow-up will continue for 3 days after surgery. Assessments will be performed at 24, 48, and 72 h for VAS pain scores at rest and with movement, cumulative sufentanil consumption via PCIA, the number of PCIA demands, and length of hospital stay. Movement-evoked pain will be assessed by asking patients to cough forcefully three times, and the highest VAS score will be recorded; this standardized maneuver will be used consistently at all assessment time points. Quality of Recovery-15 (QoR-15) scores will be assessed at 72 h postoperatively. Blinding will be assessed at 24 h postoperatively by asking participants to guess their group allocation; the proportion of correct guesses will be reported. Any perioperative adverse events related to surgery, anesthesia, or study drug administration will also be recorded.

## 3 Outcome measures

### 3.1 Baseline data

Demographic data will include body mass index (BMI), age, sex, and American Society of Anesthesiologists (ASA) physical status. Intraoperative variables will include duration of anesthesia and surgery, total sufentanil dose, cumulative remifentanil consumption, fluid administration, estimated blood loss, urine output, and hemodynamic parameters at key intraoperative time points.

### 3.2 Primary outcome

The primary outcome is the postoperative pain VAS score (0–10) with movement at 24 h postoperatively.

### 3.3 Secondary outcome

1. VAS scores at rest at 24 h, and VAS at rest and with movement at 48 h and 72 h postoperatively.
2. Total sufentanil consumption within 48 h postoperatively.
3. Number of PCIA demands and total volume consumed within 48 h postoperatively.
4. Total intraoperative opioid dosage.
5. Time to extubation.
6. Duration of postoperative hospitalization.
7. Postoperative rehabilitation quality assessment (QoR-15 score) (Supplementary Table S1).
8. Incidence of adverse events, serious adverse events, and other postoperative complications.

## 4 Statistical methods and sample size

IBM SPSS Statistics 20.0 will be used for all analyses. Normally distributed data will be presented as mean ± SD, non-normally distributed data as median (IQR), and categorical data as percentages (%). Pain scores at individual time points will be compared with an independent-samples *t*-test; repeated-measures ANOVA will evaluate quantitative variables across multiple time points between groups. Other indices will be analyzed using the *χ*^2^-test, *t*-test, or Mann–Whitney *U*-test as appropriate. Statistical significance will be set at *P* < 0.05. The proportion of correct guesses in the blinding assessment will be summarized descriptively.

This study aims to compare between-group differences in VAS scores and sufentanil consumption during the first 48 h postoperatively. The primary outcome is the difference in pain scores at 24 h, representing the peak of post-thoracotomy pain intensity (30). Based on a recent study comparing liposomal bupivacaine vs. ropivacaine for SAPB in VATS patients (23), the mean (±SD) movement-evoked VAS at 24 h was 2.1 ± 1.1 in the liposomal bupivacaine group vs. 3.9 ± 1.7 in the ropivacaine group. Using a conservative clinically meaningful difference of 1.2 (31) and a pooled SD of 1.5, with *α* = 0.05 (two-tailed) and powe*r* = 90%, 34 evaluable patients per group are required. After accounting for 10% attrition and the block size, 98 patients will be randomized, yielding approximately 88 evaluable participants and >90% power.

The primary analysis will follow an intention-to-treat (ITT) principle, including all randomized participants in their originally assigned groups. Only withdrawal of informed consent will lead to exclusion; all other post-randomization events will be treated as protocol deviations and retained in the intention-to-treat analysis. A per-protocol analysis will be performed as a sensitivity analysis. Missing primary outcome data will be handled using multiple imputation under the missing-at-random assumption.

## 5 Data collection

To minimize potential bias, the anesthetists responsible for intraoperative management and the assessors conducting postoperative evaluations will remain blinded to group allocation. This blinding ensures that the recording of intraoperative hemodynamic data and postoperative pain assessments is conducted without observer bias, thereby reducing the risk of systematic measurement bias. The number and location of thoracoscopic ports/incisions and the number and location of chest drains will be prospectively recorded; where feasible, surgical technique will be standardized across both groups. These variables will be reported descriptively and considered when interpreting the incremental benefit of SAPB. A detailed list of outcome variables is presented in Table 1.

## 6 Data monitoring and management

An independent Data and Safety Monitoring Board (DSMB), consisting of three clinicians specializing in thoracic anesthesia, perioperative care, and clinical trial methodology, will be established before patient enrollment begins. All source data will be entered into sequentially numbered, study-specific case report forms (CRFs). Completed CRFs will be stored in a secure, locked location with access restricted to the principal investigator (PI) and authorized personnel listed in the delegation log; all access events will be documented with date, time, and reason. The PI will oversee trial conduct to ensure compliance with Good Clinical Practice (GCP) and applicable regulatory requirements. De-identified data will be made available upon reasonable request after study completion.

## 7 Dissemination plans

Results will be published in peer-reviewed journals and presented at relevant scientific conferences. Findings will also be disseminated to the scientific community to inform future research and clinical practice.

## 8 Safety considerations

Inadvertent intravascular injection of liposomal bupivacaine is a potential risk. Standard practice requires aspiration before and during injection to confirm negative blood return and ensure correct needle placement. Continuous hemodynamic monitoring is used to facilitate early detection and management of adverse events. Given the off-label use of SAPB and ICNB, cumulative local anesthetic exposure will be monitored throughout the perioperative period, and early signs of local anesthetic systemic toxicity (LAST) will be assessed continuously. Resuscitation equipment and 20% lipid emulsion will be immediately available. Suspected LAST will be managed according to ASRA guidelines, including an initial intravenous bolus of 20% lipid emulsion at 1.5 mL/kg over 2–3 min, followed by a continuous infusion at 0.25 mL/kg/min, with a maximum cumulative dose of 12 mL/kg.

Adverse events (AEs) will be defined as any untoward medical occurrence after randomization, regardless of its relationship to the study intervention. Serious adverse events (SAEs) will be defined according to ICH-GCP criteria as any event resulting in death, being life-threatening, requiring inpatient hospitalization or prolongation of existing hospitalization, resulting in persistent or significant disability/incapacity, or being a congenital anomaly/birth defect. Expected events related to the regional blocks include pneumothorax, bleeding, infection, LAST, and nausea/vomiting. AEs will be collected from randomization until 72 h postoperatively through active inquiry and passive reporting. Severity will be graded as mild, moderate, or severe, and causality will be assessed as definitely related, probably related, possibly related, unlikely related, or unrelated. Should any adverse event related to liposomal bupivacaine occur, the procedure will be immediately terminated and promptly documented. All AEs will be recorded and reported, and any reactions attributable to the study intervention will be managed in accordance with institutional policy.

## 9 Discussion

This trial was designed to evaluate whether adding preoperative SAPB to ICNB provides incremental analgesic benefit in patients undergoing VATS lung resection. Both SAPB and ICNB are established regional techniques that effectively relieve postoperative pain after thoracic surgery (12, 32–34), and liposomal bupivacaine offers extended analgesia for up to 72 h (21, 35). However, evidence on their combined use remains limited, and no randomized controlled trial has directly compared the combination against ICNB alone using liposomal bupivacaine. This add-on design is clinically relevant and addresses an important evidence gap in thoracic multimodal analgesia.

The strengths of this trial include its randomized, sham-controlled design, which isolates the specific contribution of SAPB from the background analgesia provided by ICNB, and its use of a validated primary outcome (movement-evoked VAS at 24 h). Surgical and anesthetic management are standardized across groups, and a prospective blinding assessment further strengthens internal validity.

However, this study has several limitations. First, the single-center design and relatively modest sample size (98 participants) limit generalizability and are insufficient to detect rare adverse events. Second, although SAPB is performed before anesthesia induction, the gradual onset of liposomal bupivacaine and immediate transfer for anesthesia induction minimize the window for perceptible sensory changes; nevertheless, a prospective blinding assessment will be performed at 24 h postoperatively. Third, the off-label use of liposomal bupivacaine for SAPB and ICNB should be acknowledged, although published evidence supports its safety in thoracic fascial plane blocks. Finally, the sham SAPB procedure in the control group, though without therapeutic benefit and adding an invasive step, is essential for minimizing performance bias.

In summary, optimal postoperative pain control remains essential for recovery and well-being after thoracic surgery. If our findings demonstrate that combined SAPB and ICNB reduces postoperative analgesic requirements compared with sham SAPB plus ICNB, this approach may represent a more effective analgesic strategy for postoperative pain management in this patient population.
