# Supplementary material for: Effect of adding preoperative serratus anterior plane block to intercostal nerve block with liposomal bupivacaine on postoperative pain in patients undergoing video-assisted thoracoscopic lung resection: study protocol for a prospective randomized controlled trial
Source: Front Med (Lausanne). 2026 Oct 13;13:1948798. doi: 10.3389/fmed.2026.1948798 (PMC13710318; doi:10.3389/fmed.2026.1948798)
Supplement: Supplementary file 1 [file Table1.docx]

**Supplementary Material**

**Supplementary Table 1: [The quality of recovery score (QoR)-15 questionnaire.](https://www.ovid.com/image-api/imgsrv/article/i/thumb/wkmrid/JOURNAL%2Fanet%2F04.03%2F00000542-201306000-00016%2Ffigure2%2Fv%2F2024-09-28T161608Z%2Fr%2Fimage-png/width/1200.webp)**

**Date:** _ _/_ _/_ _	**ID:** _ _

**PART A**

**How have you been feeling in the last 24 hours?**

(0 to 10, where: 0=none of the time [poor] and 10=all of the time [excellent]

| **Questions** | **Score(0-10)** |
| --- | --- |
| 1. Able to breathe easily |  |
| 2. Been able to enjoy food |  |
| 3. Feeling rested |  |
| 4. Have had a good sleep |  |
| 5. Able to look after personal toilet and hygiene unaided |  |
| 6. Able to communicate with family or friends |  |
| 7. Getting support from hospital doctors and nurses |  |
| 8. Able to return work or usual home activities |  |
| 9. Feeling comfortable and in control |  |
| 10. Having a feeling of general well-being |  |

**PART B**

**Have you had any of the following in the last 24 hours?**

(10 to 0, where: 10 = none of the time [excellent] and 0 = all of the time [poor])

| **Questions** | **Scores(0-10)** |
| --- | --- |
| 11. Moderate pain |  |
| 12. Severe pain |  |
| 13. Nausea or vomiting |  |
| 14. Feeling worried or anxious |  |
| 15. Feeling sad or depressed |  |
